# Microbial community succession of cow manure and tobacco straw composting

**DOI:** 10.3389/frmbi.2024.1301156

**Published:** 2024-02-08

**Authors:** Songqing Liu, Juan Zhao, Wen-Long Feng, Zong-Jin Zhang, Yun-Fu Gu, Yan-Ping Wang

**Affiliations:** ^1^ College of Chemistry and Life Sciences, Sichuan Provincial Key Laboratory for Development and Utilization of Characteristic Horticultural Biological Resources, Chengdu Normal University, Chengdu, China; ^2^ College of Resources, Sichuan Agricultural University, Chengdu, China; ^3^ China National Tobacco Corporation Sichuan Provincial Company, Chengdu, China; ^4^ Panzhihua Branch of Sichuan Totacco Corporation, Panzhihua, China

**Keywords:** composting, cow manure, high throughput sequencing, metabolism, microbial community, tobacco straw

## Abstract

Composting livestock manure using microorganisms is a safe and resourceful practice. The continual fluctuations in physicochemical parameters during composting are intricately linked to the composition of microbial communities. This study investigated the dynamics of microbial communities during the composting of cow manure and tobacco straw using amplicon sequencing and shotgun metagenomics. The sequencing results revealed major genera such as *Sphaerobacter*, *Actinomadura*, *Thermomonospora*, *Flavobacterium*, *Bacillus*, *Hydrogenophaga*, *Pseudomonas*, *Lysinibacillus*, *Aneurinibacillus*, and *Azotobacter*. Metagenomic analysis highlighted that the phylum Proteobacteria constituted the largest proportion. Furthermore, the presence of the genus *Rhodococcus*, known to cause human and animal diseases, gradually decreased over time. These findings offer initial insights into the microbial community composition and function during cow manure and tobacco straw composting.

## 1 Introduction

In recent years, the Chinese livestock and poultry breeding industry has experienced rapid growth, with an annual production volume of animal manure reaching 3.8 billion tons (Ma et al., 2018). However, the comprehensive utilization rate of this manure remains suboptimal, posing significant ecological and environmental risks that ultimately affect human and animal health (Niu and Ju, 2017; Liu et al., 2022). Furthermore, substantial volumes of crop residues—such as wheat, corn, rice straw, and discarded tobacco leaves—which could be valuable organic fertilizers, are often regarded as waste (Yadvinder-Singh et al., 2005). Effective management of these animal manures and agricultural wastes is imperative for sustainable agriculture. Composting is broadly recognized as an effective method for disposing of agricultural and livestock waste, yielding a final product suitable for agricultural and horticultural use, aligning with sustainable strategies. Tobacco straw and cow manure composting is an effective method to reduce environmental impact. Tobacco straw, which is a byproduct during cigarette manufacturing, is disposed by burning (Yang et al., 2022). Composting, comprising aerobic composting and anaerobic digestion, is vital for treating and recycling these organic wastes. Aerobic composting, being less reliant on specialized equipment than anaerobic digestion, has proven more convenient and time-efficient (Meena et al., 2021).

Generally, composting occurs in three stages: mesophilic, thermophilic, and curing/mature phases (Papale et al., 2021). Throughout this process, aerobic microorganisms decompose organic waste into humus-like substances, enhancing soil quality as an amendment. Microorganisms metabolize organic matter, releasing energy and nutrients that aid in compost maturation. Microbial growth and reproduction are facilitated during compost maturation (Duan et al., 2020). Water-soluble small-molecule organic matter is absorbed and used by microorganisms for reproduction, whereas macromolecular organic matter is decomposed by extracellular enzymes secreted by microorganisms. Some water-soluble small-molecule organic matter is converted into substances for microbial reproduction and utilization, whereas the remainder transforms into simple inorganic substances through microbial metabolism (Zhao et al., 2017; Yu et al., 2019a; Yu et al., 2019b). Additionally, the high-temperature environment generated by microbial decomposition of organic matter in the pile eliminates weed seeds, roundworm eggs, and pathogenic bacteria in the feces (Bhattacharya and Pletschke, 2014).

The microbiome plays a critical role in composting. The community structure of microorganisms undergoes dynamic changes during composting, influenced by factors such as proportions of composting materials, and composting methods and conditions (Wang et al., 2017; Li et al., 2020). Understanding microbial communities throughout composting is crucial for system comprehension and optimizing compost quality. Additionally, the intricate actions of numerous microorganisms are directly influenced by various environmental factors in composting, including temperature, moisture, carbon/nitrogen ratio, oxygen levels, and pH (Insam et al., 2010). The composition and dynamics of microbial communities in composts have been explored using both culture-dependent and culture-independent methods (Chow et al., 2014; Petersen et al., 2015). Nonetheless, our understanding of microbial community structures, especially fungal communities, in specific crop and livestock waste composting processes remains limited because of the complexity of microbial interactions and the incomplete nature of current studies. Therefore, we aimed to delineate changes in microbial communities during composting using high-throughput sequencing to confirm the metabolic pathways critical in the composting process.

## 2 Materials and methods

### 2.1 Experimental design and sample collection

In March 2020, three natural composting piles containing cow manure and tobacco straw at a ratio of 4:1 were prepared in Panzhihua, Sichuan, China. For the composting piles, which were approximately 2.5 m × 1.5 m × 1.5 m (length × width × height), tobacco straw was used as the bulking material. These piles maintained approximately 65% moisture content and a 32:1 C/N ratio. The characteristics of the raw materials are itemized in **Table 1**. Before the mature state, three artificial turnings were performed on days 9, 15, 20, and 26, as the compost temperature reached 65°C for 27 days. Sub-samples were collected from nine different points at three depths (30 cm, 60 cm, and 120 cm from the top) of the composting piles on days 0, 9, 15, 20, and 26, representing initial, mesophilic, thermophilic, cooling, and maturation phases, respectively. The sub-samples were mixed and divided into three portions. One portion was stored at −80°C for DNA extraction, one portion was stored at 4°C for the measurement of ammonium and nitrate, and the remaining portion was air-dried for physicochemical analyses.

**Table 1:** The physicochemical characteristics of the raw materials.

	pH	Moisture content (%)	Total organic carbon (g/kg)	TN (g/kg)	C/N	NO_3_^−^-N	NH_4_^+^-N
Cow manure	8.98 ± 0.53a	69.48 ± 5.67a	385.0 ± 12.06a	17.30 ± 0.517a	22.29 ± 0.69a	102.1 ± 10.2	988.4 ± 21.6
Tobacco straw	7.25 ± 0.47b	13.57 ± 3.89b	336.0 ± 26.83a	5.26 ± 0.507b	64.42 ± 1.12b	nd	nd

Data are mean ± SE (n = 3). Different lowercase letters in a column indicate statistically significant differences at p < 0.05.

TN, total nitrogen; TN, total nitrogen; C/N, the ratio of total organic carbon to TN. "n.d" means "Not determined.

### 2.2 Physicochemical parameter analysis

Digital thermometers were used to measure temperature near the composting piles and at the surface, core, and bottom of the composting piles daily. pH was measured after shaking fresh samples in water at a 1:10 (w/v) ratio at 120 r/min for 60 minutes, and moisture content was determined by oven-drying to a constant weight at 105°C (Abid and Sayadi, 2006). The total organic carbon (TC) content was determined using the dry combustion method. Total nitrogen (TN) content was assessed using the Kjeldahl method (Kimberly and Roberts, 1905; Abad et al., 2002). Ammonium (NH_4_^+^-N) and nitrate (NO_3_^−^-N) were extracted using 2 mol/L KCl and analyzed using a dual-channel flow analyzer (AA3, Seal Analytical, Norderstedt, Germany) (Ren et al., 2023).

### 2.3 Amplicon and metagenomic sequencing

DNA was extracted as described earlier (Liu et al., 2011). The extracted DNA underwent purification using a DNA gel purification kit (Omega, Norcross, GA, USA) as per the manufacturer’s instructions. DNA quality was verified through electrophoresis in a 1.0% agarose gel, and concentration was determined using a spectrophotometer (NanoDrop 2000, Thermo Fisher Scientific, Waltham, MA, USA).

The primers 515F (5′-GTGCCAGCMGCCGCGGTAA-3′) and 806R (5′-GGACTACVSGGG-TATCTAAT-3′) incorporating adapter and barcode sequences (Caporaso et al., 2012) were used to amplify the 16S rRNA gene V4 hypervariable region. PCR amplification was performed in a 25.0-µL reaction solution comprising 12.5 µL Taq-HS PCR Forest Mix, 0.2 µL of each primer, 1.0 µL template DNA, and 11.1 µL ddH_2_O. Purified PCR products with concentrations exceeding 10 ng/µL and OD 260/OD 280 ≈ 1.8 were sequenced on the Illumina MiSeq platform at Shanghai Personalbio Technology Co., Ltd. (Shanghai, China). Details of the data analysis are provided in the **Supplementary Material**.

Amplicon sequence reads were processed using QIIME2 v2019.4 (Bokulich et al., 2018) following official tutorials (https://docs.qiime2.org/2019.4/tutorials/). Initially, raw sequence data underwent demultiplexing using the demux plugin, followed by primer cutting using the QIIME 2 Cutadapt plugin (Martin, 2011). Quality filtering involved QIIME’s split_libraries_fastq.py script, discarding reads with Phred quality scores <29 and consecutive, high-quality base calls less than 90% of the read’s length. Removal of chimeric, singleton, and non-bacterial sequences, such as chloroplast and mitochondrial sequences, was conducted using the debulr plugin (Schuler et al., 2016). Non-singleton amplicon sequence variants (ASVs) were aligned using mafft (Katoh et al., 2002). Subsequently, after rarefaction, an estimation of the Shannon diversity index was performed using the diversity plugin in QIIME2. Taxonomy was assigned to the ASVs *via* the classify-sklearn naïve Bayes taxonomy classifier in the feature-classifier plugin against the SILVA database (Pelin Yilmaz et al., 2014). Alpha diversity metrics were used to summarize the microbial community structure concerning richness, evenness, or both (Willis, 2019). Metrics included Chao1 (Chao, 1984), observed species, Faith’s PD (Faith, 1992), Shannon (Simpson, 1949), Simpson, Pielou’s evenness (Pielou, 1966), and Good’s coverage (Good, 1953). The Shannon diversity index (*H*) was calculated using the “diversity” function in the Vegan package (Oksanen et al., 2015).

For metagenomic sequencing, DNA underwent fragmentation into approximately 400-bp fragments using an ultrasonic disruptor (Covaris M220, Gene Company Limited, Hong Kong, China), followed by Illumina library construction using a NEXTFLEX Rapid DNA-Seq Library Prep kit (PerkinElmer, Waltham, MA, USA). Sequencing occurred on an Illumina PE150 instrument (Illumina, San Diego, CA, USA). Quality filtering of the data was conducted through a laboratory information management system (LIMS) within the open-source Galaxy platform (https://usegalaxy.org/). Clean reads from the metagenomic dataset were assembled into contigs using the SOAP denovo assembler (Li et al., 2010). Subsequently, the contigs were annotated using the MGRAST (metagenomics Rapid Annotation using Subsystem Technology, Version 4.0) platform in the public project id2017chunjie (http://metagenomics.anl.gov/) with the Kyoto Encyclopedia of Genes and Genomes (KEGG) Orthology (KO) database. Protein sequences translated from open reading frames (ORFs) were aligned with the National Center for Biotechnology Information (NCBI) database using the Basic Local Alignment Search Tool (BLAST) with an E-value < 10^−5^ (Ma et al., 2016). Mapping of sequences to KEGG pathways was performed by importing the BLAST results into MEGAN, using the “KEGG viewer” module (He et al., 2016). To assess the carbon utilization potential within the microbial communities during cow manure and tobacco straw composting, non-redundant genes were cross-referenced with the carbohydrate-active enzyme database (CAZy) using DIAMOND software (e < 1e−5) (Buchfink et al., 2015). Proteins exhibiting the highest sequence similarity underwent screening and were further subjected to CAZy analysis, searching against sequence libraries encompassing glycoside hydrolases (GHs), auxiliary activities (AAs), carbohydrate-binding modules (CBMs), glycosyltransferases (GTs), polysaccharide lyases (PLs), and carbohydrate esterases (CEs).

### 2.4 Statistical analysis

Differences in physicochemical properties, bacterial Shannon diversity index, and 16S rRNA gene abundance were tested using one-way ANOVA. Additionally, bacterial community structure was visualized *via* non-metric multidimensional scaling (NMDS) using the Bray–Curtis dissimilarity matrices in the Vegan package (Oksanen et al., 2020).

## 3 Results and discussion

### 3.1 Physicochemical properties

**Figure 1:**
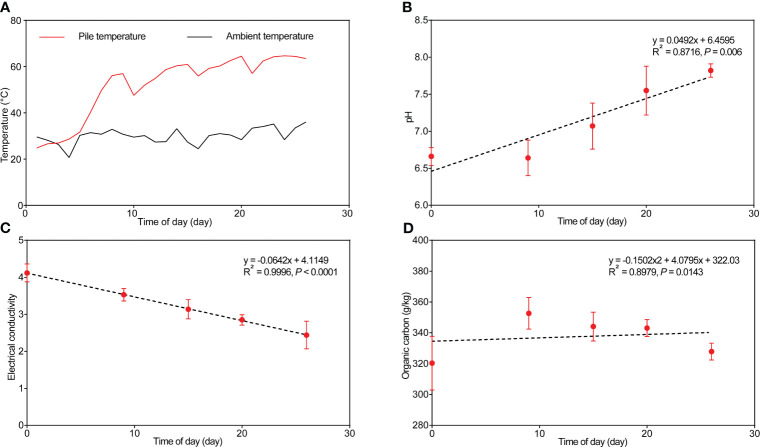
Changes in temperature **(A)**, pH **(B)**, electrical conductivity **(C)**, and organic carbon content **(D)** in the cow manure and tobacco straw compost during the composting process.

Temperature stands as a crucial indicator throughout composting, reflecting the composting process and alterations in microbial activities (Zheng et al., 2015). The cow manure and tobacco straw composting mixture’s temperature rapidly increased to 50°C within 1 week (**Figure 1A**, **Supplementary Table S1**). Sustained high temperatures persisted for the subsequent 20 days, reaching 63°C on the 20th day, increasing the average temperature of the entire process by 20°C compared with the environment, significantly enhancing the fermentation process. The degradation of organic matter generates thermal energy, especially during the initial and thermophilic phases (Lu et al., 2009).

Initially, the moisture content of the compost was at approximately 59.7% and gradually decreased within the first 15 days of composting (**Supplementary Table S1**). Water evaporation results from heat generated by microbial reactions during composting, reducing moisture content in the compost pile (Miller and Finstein, 1985). Generally, the pH value correlated positively with composting time (**Figure 1B**, *p* = 0.006), increasing from the initial 6.5 to the final 7.82. Electrical conductivity reduced from 4.12 mS/cm to 2.44 mS/cm during composting (**Figure 1C**) and correlated negatively with composting time (*p* < 0.0001).

The overall nutrient content change aligned with the variations in TN, total phosphorus (TP), and total potassium (TK), initially increasing and then decreasing, finally peaking at day 15 (**Supplementary Table S1**). Organic carbon content was the highest on day 9, followed by a decrease until the composting’s conclusion (**Figure 1D**). The changes in NH_4_^+^-N and NO_3_^−^-N concentrations exhibited reverse trends (**Supplementary Table S1**). NH_4_^+^-N concentration peaked at 60.6 mg/kg, greater than its level during the primary stage (52.1 mg/kg) of composting. The C/N ratio in the organic matter used for composting influences microbial fermentation and decomposition. A high C/N ratio slows microbial decomposition and consumes available N in the soil. In agreement with Duan et al. (2020), in our study, the C/N ratio was the highest on the ninth day of composting (26.5) and the lowest on the 26th day (23.1) (**Supplementary Table S1**).

### 3.2 Taxonomic diversity and abundance of the microbial communities

**Figure 2:**
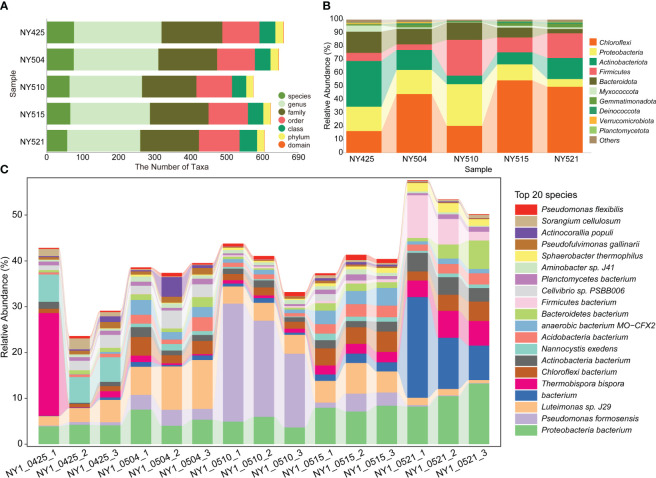
The distribution of microbial taxa in the cow manure and tobacco straw compost during the composting process. **(A)** The numbers of identified taxa. **(B)** The relative abundances of major phyla in the 16S rRNA (NY) gene amplicon data. **(C)** The relative abundances of major species in the metagenomic data (NY1).

**Figure 3:**
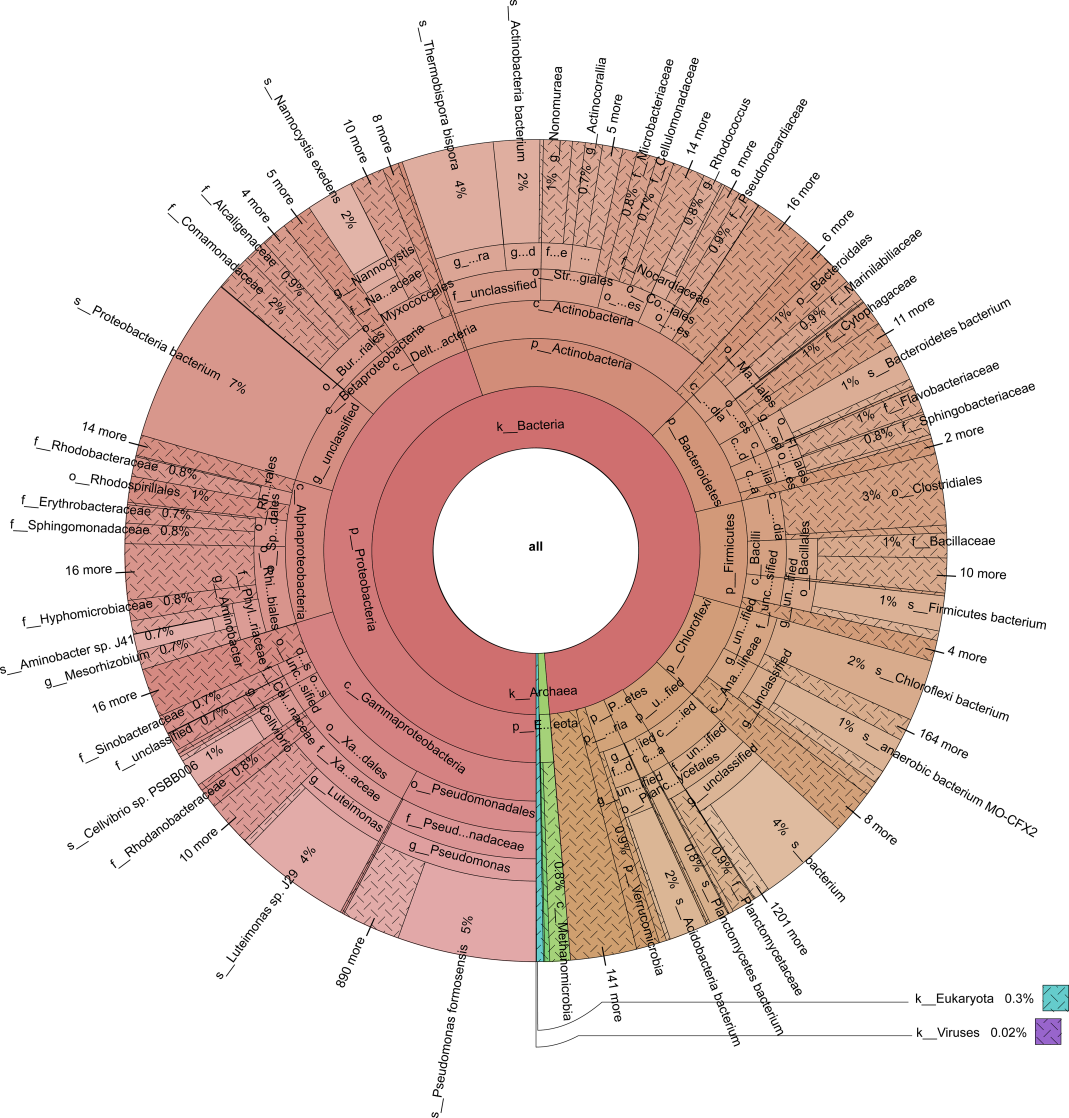
The distribution of taxa in the cow manure and tobacco straw compost metagenome.

The bacterial community structure changed during composting (**Figure 2A**). The species count was the highest on day 0. The relative abundance of *Chloroflexi* was the highest on days 9, 20, and 26. The relative abundance of *Pseudomonas formosensis* (Proteobacteria) was the highest on day 15 (**Figures 2B, C**). Proteobacteria were the predominant bacteria during all composting stages (**Figures 2C**, **3**).

**Figure 4:**
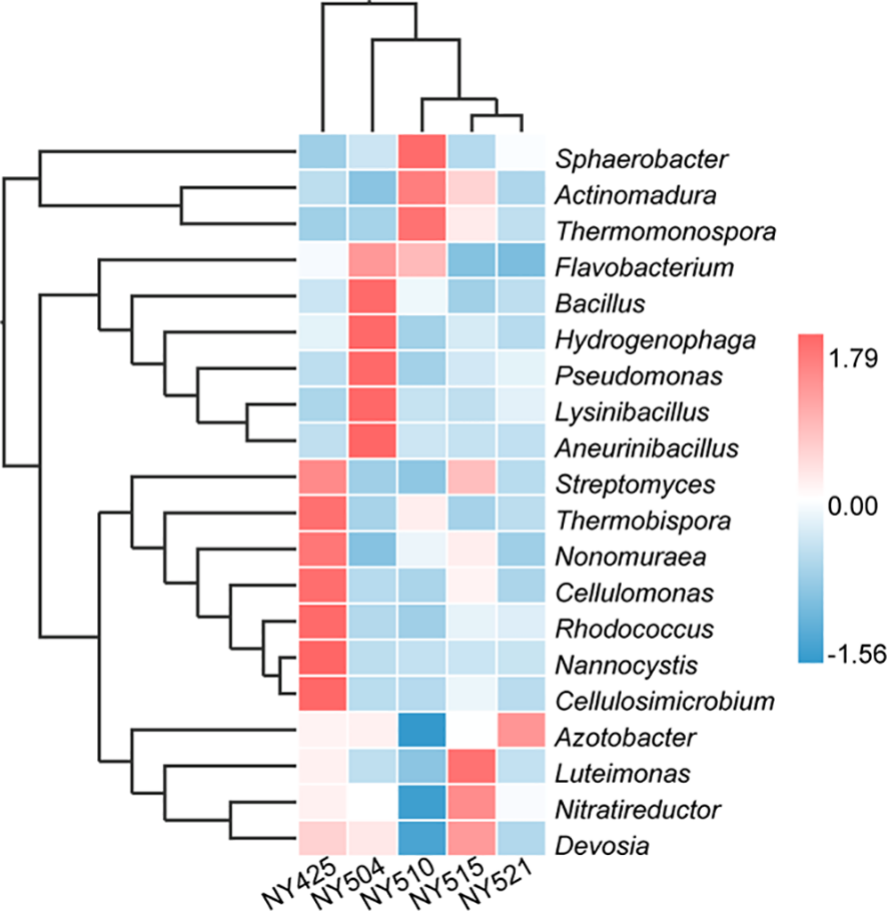
Relative abundances of the 20 most abundant genera in the cow manure and tobacco straw compost during the composting process.

**Figure 5:**
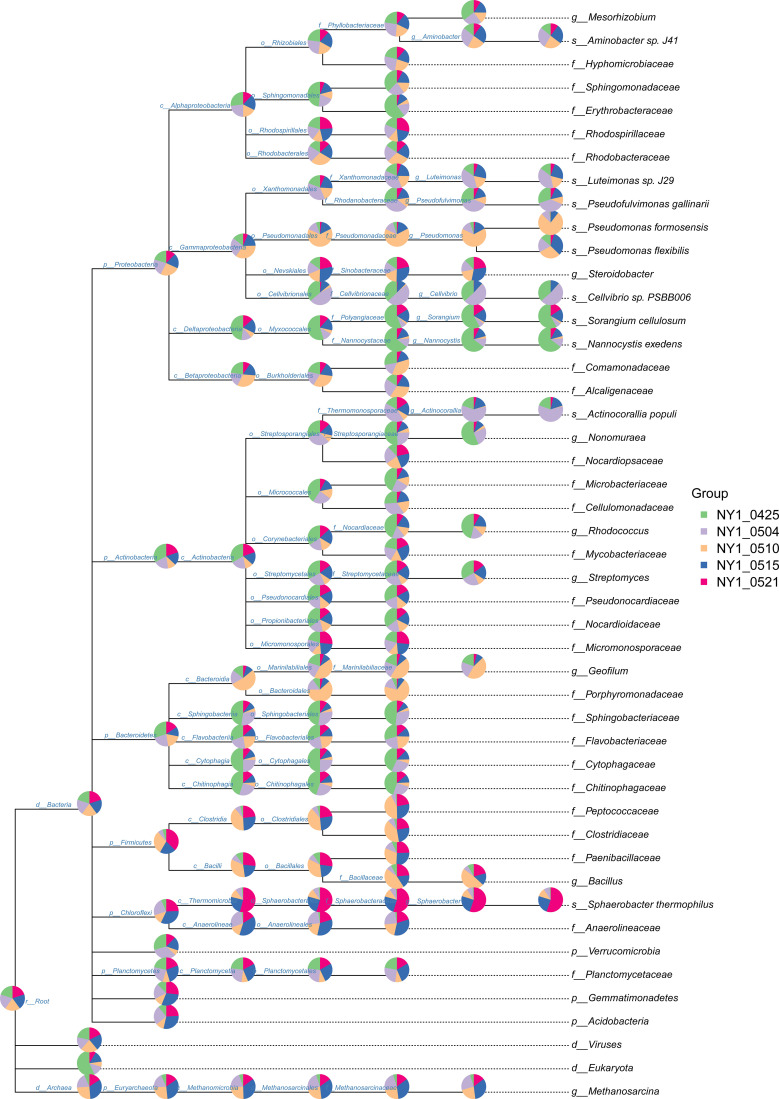
Taxonomic composition of the cow manure and tobacco straw compost metagenome during the composting process.

Bacterial community composition constantly evolved during composting, with the relative abundances of genera such as *Sphaerobacter*, *Actinomadura*, *Thermomonospora*, *Flavobacterium*, *Bacillus*, *Hydrogenophaga*, *Pseudomonas*, *Lysinibacillus*, *Aneurinibacillus*, *Azotobacter*, *Luteimonas*, *Nitratireductor*, *Devosia*, and *Streptomyces* peaking at high-temperature stages (**Figure 4**). Notably, genera involved in lignin degradation, such as *Thermopolyspora* and *Sphaerobacter* (Shivlata and Satyanarayana, 2015; Kwon et al., 2019), became prominent. Lignin degradation secreted laccase and lignin peroxidase to produce polyphenols or phenol, which is a soil improvement substance that is returned to the field (Zhao et al., 2021). Metagenomic analysis further revealed an increase in *Sphaerobacter thermophilus* with the progress of composting (**Figure 5**). The relative abundance of genus *Rhodococcus*, including the pathogen *Rhodococcus equi* affecting animals and humans (Prescott, 1991), gradually decreased during composting. During the composing process, the high temperature as the main abiotic stress is critical for mutualistic interactions of microbial communities (Zhao et al., 2023). As our results suggest, the bacteria were increased on the 27th day at 63°C, indicating that mutualistic interactions of bacteria exist in cow manure and tobacco straw composting (**Figure 2C**).

**Figure 6:**
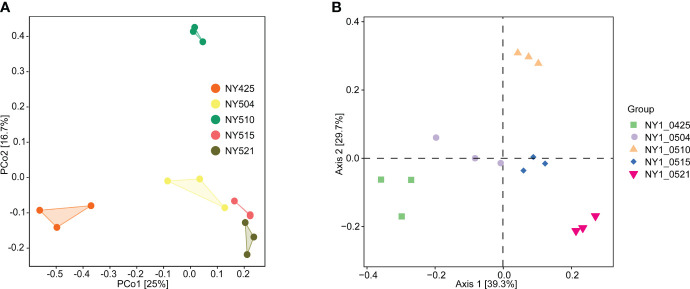
Bray–Curtis dissimilarity-based beta diversity in the cow manure and tobacco straw compost during the composting process. 16S rRNA amplicon sequencing **(A)** and metagenome sequencing **(B)** data.

Chao1 index and the number of observed species were lower from day 9 onward than on day 0 (**Table 2**), potentially because of environmental changes during composting, e.g., increasing compost temperature (**Figure 1**). Similarly, Good’s coverage, Pielou’s evenness, Shannon, and Simpson indices were generally higher in the initial phase than in the later stages with higher compost temperature, indicating decreasing community richness and diversity as composting progressed. In the Bray–Curtis dissimilarity-based principal coordinates analysis (PCoA), day 0 samples differed from the other samples along axis 1 (**Figure 6**), indicating differences in community composition.

**Table 2:** Alpha diversity of the bacterial communities in the cow manure and tobacco straw compost during the composting process.

	Chao1	Goods coverage	Observed species	Pielou’s e	Shannon	Simpson
Day 0	3,777.753 ± 519.213	0.982 ± 0.012	3,590.167 ± 327.196	0.802 ± 0.027	9.464 ± 0.346	0.990 ± 0.004
Day 9	3,643.557 ± 734.742	0.983 ± 0.006	3,509.900 ± 688.792	0.754 ± 0.041	8.868 ± 0.698	0.976 ± 0.014
Day 15	3,539.700 ± 279.487	0.981 ± 0.01	3,333.533 ± 265.451	0.751 ± 0.038	8.789 ± 0.442	0.983 ± 0.005
Day 20	3,446.143 ± 338.961	0.978 ± 0.009	3,161.867 ± 276.931	0.700 ± 0.036	8.138 ± 0.415	0.957 ± 0.012
Day 26	3,410.690 ± 399.36	0.981 ± 0.011	3,186.200 ± 214.284	0.710 ± 0.057	8.262 ± 0.633	0.940 ± 0.034

### 3.3 Functional profiles of metagenome

**Figure 7:**
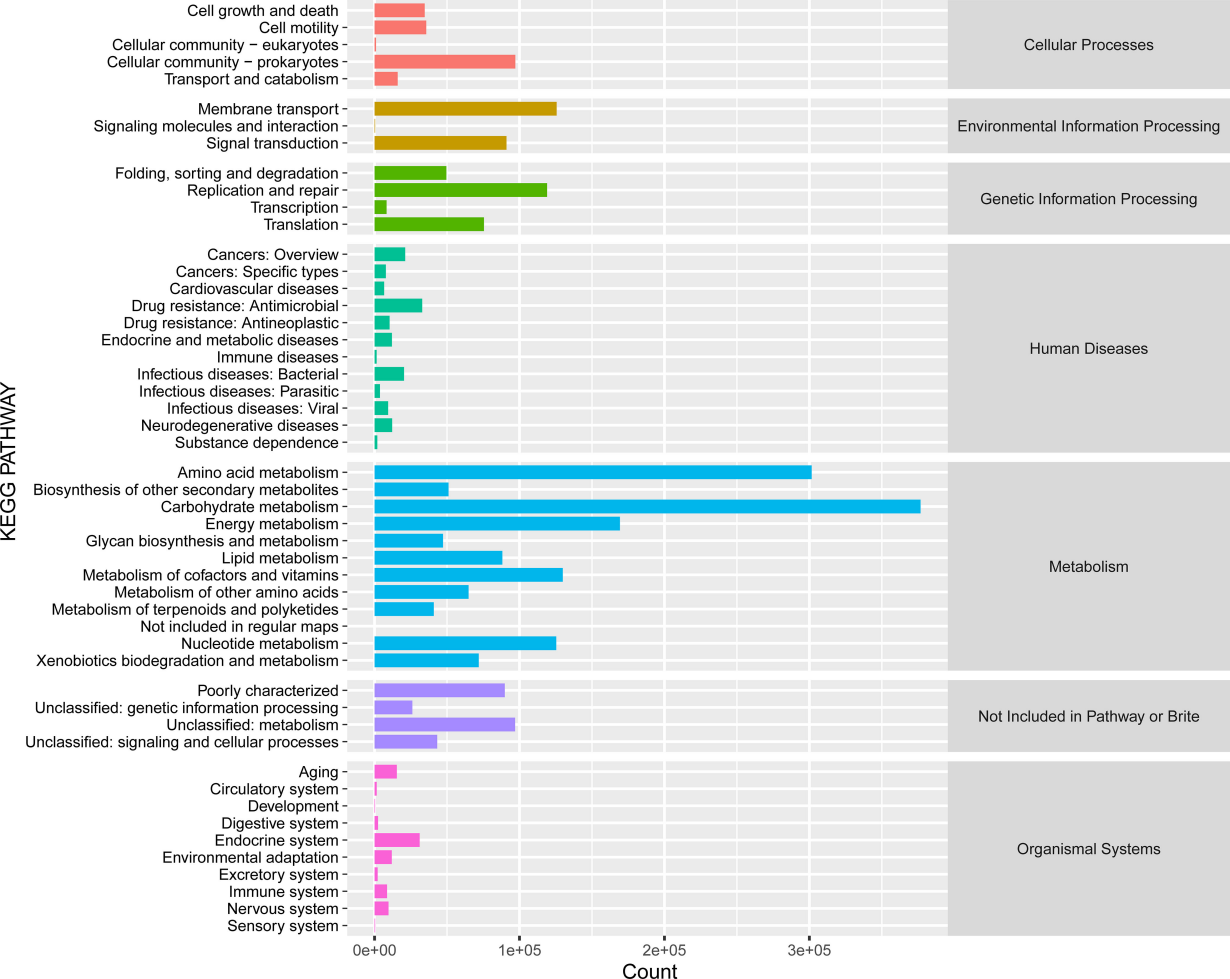
KEGG functional categories in the cow manure and tobacco straw compost metagenome. KEGG, Kyoto Encyclopedia of Genes and Genomes.

**Figure 8:**
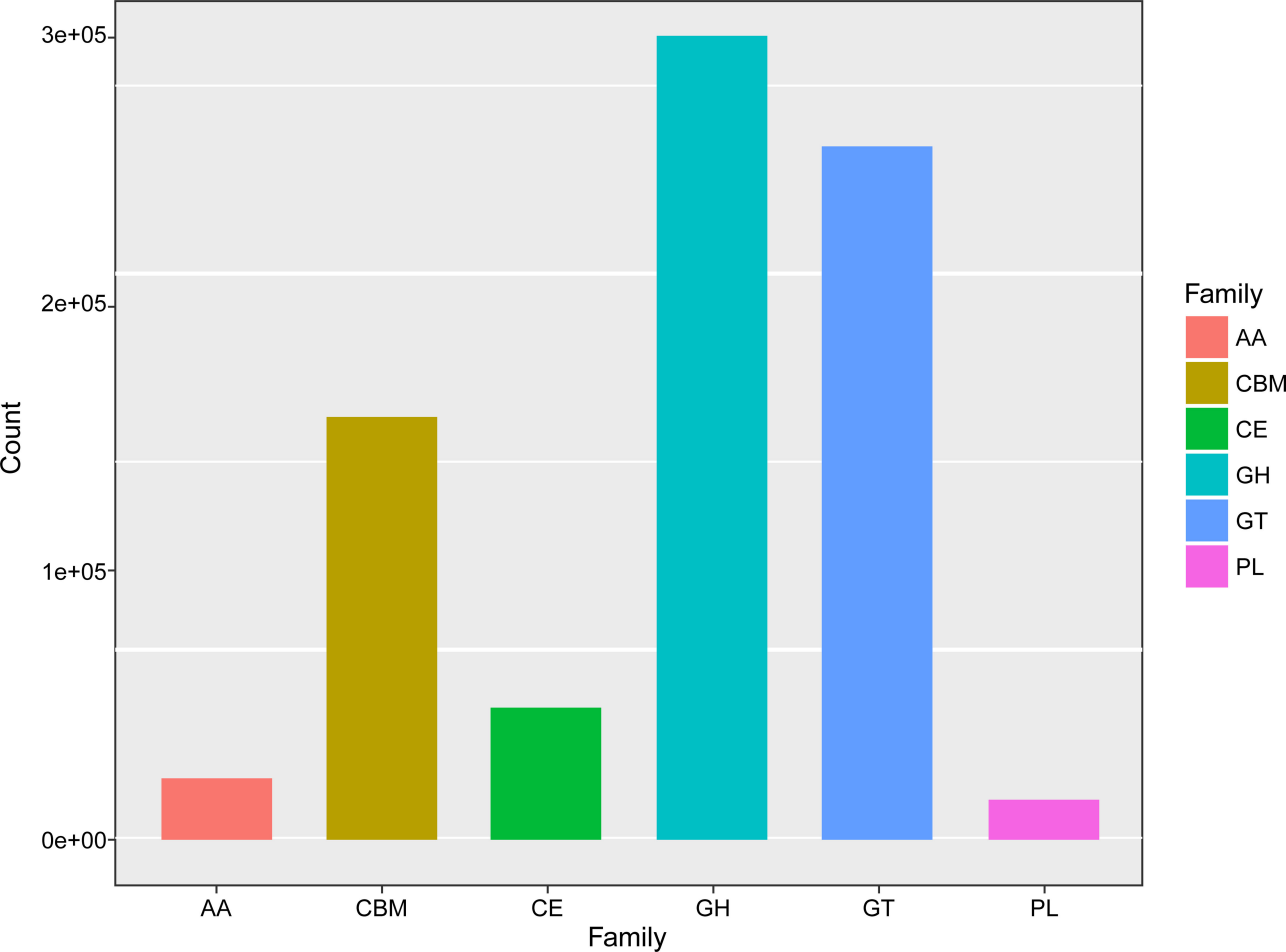
The number of genes assigned to carbohydrate-active enzymes (CAZymes) families in the cow manure and tobacco straw compost metagenome.

The KEGG category analysis revealed that carbohydrate metabolism (14.49% of all KEGG categories), amino acid metabolism (11.60%), energy metabolism (6.51%), and metabolism of cofactors and vitamins (4.99%) were the most abundant categories (**Figure 7**). Comparative KEGG analysis with lignocellulose-degrading consortia from rainforest compost, apple pomace-adapted compost (Zhou et al., 2017), and rice straw-adapted compost (Reddy et al., 2013) demonstrated similar metabolic patterns, notably in carbohydrate metabolism and amino acid transport and metabolism. A total of 799,816 genes were assigned to different carbohydrate-active enzymes (CAZymes) families (298,588 GHs, 257,520 GTs, 14,848 PLs, 22,808 AAs, 49,041 CEs, and 157,011 CBMs) across all the compost samples (**Figure 8**). These findings suggest that several functional capacities, particularly in carbohydrate metabolism, were enriched within the cow manure and tobacco straw compost microbial community.

## 4 Conclusions

Most physicochemical parameters exhibited minor variations during cow manure and tobacco straw composting. Our results indicated slight fluctuations in TN, TP, TK, total organic carbon, NO_3_^−^-N, NH_4_^+^-N, C/N, and overall nutrient levels. pH significantly increased with composting time, whereas conductivity displayed a reversed trend. The diversity and abundance of microbial communities underwent significant changes throughout the composting process. High-throughput 16S rRNA gene amplicon sequencing revealed dominant genera during composting, including *Sphaerobacter*, *Actinomadura*, *Thermomonospora*, *Flavobacterium*, *Bacillus*, *Hydrogenophaga*, *Pseudomonas*, *Lysinibacillus*, *Aneurinibacillus*, *Azotobacter*, *Luteimonas*, *Nitratireductor*, *Devosia*, and *Streptomyces*. Metagenomic data identified Proteobacteria as the predominant bacterium across all compost samples. These findings contribute to a deeper understanding of microbial community succession in cow manure and tobacco straw composting under natural conditions.

## Data availability statement

The metagenomic sequence data have been deposited to the NCBI Sequence Read Archive with Accession PRJNA1047733.

## Author contributions

SL: Formal analysis, Methodology, Visualization, Writing – original draft. JZ: Data curation, Formal analysis, Software, Visualization, Writing – original draft. W-LF: Data curation, Writing – original draft. ZZ: Data curation, Writing – original draft. Y-FG: Project administration, Supervision, Writing – review & editing. Y-PW: Conceptualization, Project administration, Supervision, Writing – original draft, Writing – review & editing.

## References

[B1] Abad M. Noguera P. PuChades R. Maquieira A. Noguera V. (2002). Physico-chemical and chemical properties of some coconut coir dusts for use as a peat substitute for containerised ornamental plants. Bioresource Technol. 82, 241–245. doi: 10.1016/s0960-8524(01)00189-4 11991072

[B2] Abid N. Sayadi S. (2006). Detrimental effects of olive mill wastewater on the composting process of agricultural wastes. Waste Manage. (New York N.Y.) 26, 1099–1107. doi: 10.1016/j.wasman.2005.06.015 16181778

[B6] Bhattacharya A. Pletschke B. I. (2014). *Thermophilic Bacilli* and their enzymes in composting. Compost. Sustain. Agric. 3, 103–124. doi: 10.1007/978-3-319-08004-8_6

[B7] Bokulich N. A. Kaehler B. D. Rideout J. R. Dillon M. Bolyen E. Knight R.. (2018). Optimizing taxonomic classification of marker-gene amplicon sequences with QIIME 2’s q2-feature-classifier plugin. Microbiome 6, 90. doi: 10.1186/s40168-018-0470-z 29773078 PMC5956843

[B9] Buchfink B. Xie C. Huson D. H. (2015). Fast and sensitive protein alignment using DIAMOND. Nat. Methods 12, 59–60. doi: 10.1038/nmeth.3176 25402007

[B10] Caporaso J. G. Lauber C. L. Walters W. A. Berg-Lyons D. Huntley J. Fierer N.. (2012). Ultra-high-throughput microbial community analysis on the Illumina HiSeq and MiSeq platforms. ISME J. 6, 1621–1624. doi: 10.1038/ismej.2012.8 22402401 PMC3400413

[B11] Chao A. (1984). Nonparametric estimation of the number of classes in a population. Scandinavian J. Stat 11, 265–270.

[B12] Chow W. S. Tan S. G. Ahmad Z. Chia K. H. Lau N.-S. Sudesh K. (2014). Biodegradability of epoxidized soybean oil based thermosets in compost soil environment. J. Polymers Environ. 22, 140–147. doi: 10.1007/s10924-013-0615-x

[B13] Duan M. Zhang Y. Zhou B. Qin Z. Wu J. Wang Q.. (2020). Effects of Bacillus subtilis on carbon components and microbial functional metabolism during cow manure-straw composting. Bioresource Technol. 303, 122868. doi: 10.1016/j.biortech.2020.122868 32032936

[B14] Faith D. P. (1992). Conservation evaluation and phylogenetic diversity. Biol. Conserv. 61, 1–10. doi: 10.1016/0006-3207(92)91201-3

[B15] Good I. J. (1953). The population frequencies of species and the estimation of population parameters. Biometrika 40, 237–264. doi: 10.1093/biomet/40.3-4.237

[B16] He S. Ding L.-L. Xu K. Geng J.-J. Ren H.-Q. (2016). Effect of low temperature on highly unsaturated fatty acid biosynthesis in activated sludge. Bioresource Technol. 211, 494–501. doi: 10.1016/j.biortech.2016.03.069 27035483

[B17] Insam H. Franke-Whittle I. Goberna M. (2010). “Microbes in aerobic and anaerobic waste treatment,” in Microbes at Work: From Wastes to Resources. Eds. Insam H. Franke-Whittle I. Goberna M. (Berlin, Heidelberg: Springer Berlin Heidelberg), 1–34. doi: 10.1007/978-3-642-04043-6_1

[B18] Katoh K. Misawa K. Kuma K. Miyata T. (2002). MAFFT: a novel method for rapid multiple sequence alignment based on fast Fourier transform. Nucleic Acids Res. 30, 3059–3066. doi: 10.1093/nar/gkf436 12136088 PMC135756

[B19] Kimberly A. E. Roberts M. G. (1905). A method for the direct determination of organic nitrogen by the kjeldahl process. Public Health papers Rep. 31, 109–122. PMC2222302 19601246

[B20] Kwon S. Lee J. H. Kim C. M. Ha H. J. Lee S. H. Lee C. S.. (2019). Structural insights into the enzyme specificity of a novel ω-transaminase from the thermophilic bacterium *Sphaerobacter thermophilus* . J. Struct. Biol. 208, 107395. doi: 10.1016/j.jsb.2019.09.012 31560999

[B22] Li Y. Liu Y. Yong X. Wu X. Jia H. Wong J. W. C.. (2020). Odor emission and microbial community succession during biogas residue composting covered with a molecular membrane. Bioresource Technol. 297, 122518. doi: 10.1016/j.biortech.2019.122518 31812915

[B21] Li R. Zhu H. Ruan J. Qian W. Fang X. Shi Z.. (2010). *De novo* assembly of human genomes with massively parallel short read sequencing. Genome Res. 20, 265–272. doi: 10.1101/gr.097261.109 20019144 PMC2813482

[B23] Liu J. Xu X.-h. Li H.-t. Xu Y. (2011). Effect of microbiological inocula on chemical and physical properties and microbial community of cow manure compost. Biomass Bioenergy 35, 3433–3439. doi: 10.1016/j.biombioe.2011.03.042

[B24] Liu Z. Wei Y. Li J. Ding G. C. (2022). Integrating 16S rRNA amplicon metagenomics and selective culture for developing thermophilic bacterial inoculants to enhance manure composting. Waste Manage. (New York N.Y.) 144, 357–365. doi: 10.1016/j.wasman.2022.04.013 35436715

[B25] Lu Y. Wu X. Guo J. (2009). Characteristics of municipal solid waste and sewage sludge co-composting. Waste Manage. 29, 1152–1157. doi: 10.1016/j.wasman.2008.06.030 18783931

[B27] Ma S. Fang C. Sun X. Han L. He X. Huang G. (2018). Bacterial community succession during pig manure and wheat straw aerobic composting covered with a semi-permeable membrane under slight positive pressure. Bioresource Technol. 259, 221–227. doi: 10.1016/j.biortech.2018.03.054 29558720

[B26] Ma J. Wang Z. Li H. Park H.-D. Wu Z. (2016). Metagenomes reveal microbial structures, functional potentials, and biofouling-related genes in a membrane bioreactor. Appl. Microbiol. Biotechnol. 100, 5109–5121. doi: 10.1007/s00253-016-7312-3 26816093

[B28] Martin M. (2011). Cutadapt removes adapter sequences from high-throughput sequencing reads. EMBnet. journal 17, 10–12. doi: 10.14806/ej.17.1.200

[B29] Meena A. L. Karwal M. Kj R. Narwal E. (2021). Aerobic composting versus Anaerobic composting: Comparison and differences. Food Sci. 2, 23–26.

[B30] Miller F. Finstein M. (1985). Materials balance in the composting of wastewater sludge as affected by process control strategy. Water pollut. Control Fed. 57, 122–127. doi: 10.2307/25042542

[B31] Niu X.-S. Ju X.-T. (2017). Organic fertilizer resources and utilization in China. J. Plant Nutr. Fertil. 23, 1462–1479. doi: 10.11674/zwyf.17430

[B32] Oksanen J. Blanchet F. G. Friendly M. Kindt R. Legendre P. McGlinn D.. (2020). vegan community ecology package version 2.5-7 R Package Version 2.5-7 November 2020. https://cran.rproject.org/web/packages/vegan/index.html.

[B33] Oksanen J. Blanchet F. G. Kindt R. Legendre P. Minchin P. O’Hara B.. (2015). Vegan: Community Ecology Package. R Package Version 2.2-1, Vol. 2. 1–2.

[B34] Papale M. Romano I. Finore I. Lo Giudice A. Piccolo A. Cangemi S.. (2021). Prokaryotic diversity of the composting thermophilic phase: the case of ground coffee compost. Microorganisms 9, 218. doi: 10.3390/microorganisms9020218 PMC7911569 33494462

[B35] Petersen C. Saebelfeld M. Barbosa C. Pees B. Hermann R. J. Schalkowski R.. (2015). Ten years of life in compost: temporal and spatial variation of North German Caenorhabditis elegans populations. Ecol. Evol. 5, 3250–3263. doi: 10.1002/ece3.1605 26380661 PMC4569023

[B37] Pielou E. C. (1966). The measurement of diversity in different types of biological collections. J. Theor. Biol. 13, 131–144. doi: 10.1016/0022-5193(66)90013-0

[B38] Prescott J. F. (1991). Rhodococcus equi: an animal and human pathogen. Clin. Microbiol. Rev. 4, 20–34. doi: 10.1128/cmr.4.1.20 2004346 PMC358176

[B39] Reddy A. P. Simmons C. W. D’Haeseleer P. Khudyakov J. Burd H. Hadi M.. (2013). Discovery of microorganisms and enzymes involved in high-solids decomposition of rice straw using metagenomic analyses. PloS One 8, e77985. doi: 10.1371/journal.pone.0077985 24205054 PMC3808287

[B40] Ren Y. M. Hou Z. J. Su T. Lin Z. R. Liu A. Q. Cai L. P. (2023). Characteristics and correlation of soil low-molecular-weight organic acids and nutrients in four plantations in red soil area of south China. Int. J. Environ. Sci. Technol. 20, 6339–6350. doi: 10.1007/s13762-022-04319-0

[B41] Schuler C. J. Hirsch M. Harmeling S. Scholkopf B. (2016). Learning to deblur. IEEE Trans. Pattern Anal. Mach. Intell. 38, 1439–1451. doi: 10.1109/tpami.2015.2481418 26415157

[B42] Shivlata L. Satyanarayana T. (2015). Thermophilic and alkaliphilic Actinobacteria: biology and potential applications. Front. Microbiol. 6. doi: 10.3389/fmicb.2015.01014 PMC4585250 26441937

[B43] Simpson E. H. (1949). Measurement of diversity. Nature 163, 688–688. doi: 10.1038/163688a0

[B45] Wang Q. Awasthi M. K. Ren X. Zhao J. Li R. Wang Z.. (2017). Comparison of biochar, zeolite and their mixture amendment for aiding organic matter transformation and nitrogen conservation during pig manure composting. Bioresource Technol. 245, 300–308. doi: 10.1016/j.biortech.2017.08.158 28898824

[B46] Willis A. D. (2019). Rarefaction, alpha diversity, and statistics. Front. Microbiol. 10. doi: 10.3389/fmicb.2019.02407 PMC6819366 31708888

[B48] Yadvinder-Singh S. Bijay S. Jagadish T. (2005). Crop residue management for nutrient cycling and improving soil productivity in rice-based cropping systems in the tropics. Adv. Agron. - ADVAN Agron. 85, 269–407. doi: 10.1016/S0065-2113(04)85006-5

[B49] Yang K. Jiang Y. Wang J. Cai X. Wen Z. Qiu Z.. (2022). Tobacco straw biochar improved the growth of Chinese cherry (Prunus pseudocerasus) via altering plant physiology and shifting the rhizosphere bacterial community. Scientia Hortic. 303, 111244. doi: 10.1016/j.scienta.2022.111244

[B36] Yilmaz P. Parfrey L. W. Yarza P. Gerken J. Pruesse E. Quast C.. (2014). The SILVA and “All-species Living Tree Project (LTP)” taxonomic frameworks. Nucleic Acids Res. 42 (D1), 643–648. doi: 10.1093/nar/gkt1209 24293649 PMC3965112

[B50] Yu H. Xie B. Khan R. Shen G. (2019a). The changes in carbon, nitrogen components and humic substances during organic-inorganic aerobic co-composting. Bioresource Technol. 271, 228–235. doi: 10.1016/j.biortech.2018.09.088 30273826

[B51] Yu H. Zhao Y. Zhang C. Wei D. Wu J. Zhao X.. (2019b). Driving effects of minerals on humic acid formation during chicken manure composting: Emphasis on the carrier role of bacterial community. Bioresource Technol. 294, 122239. doi: 10.1016/j.biortech.2019.122239 31610491

[B53] Zhao Y. Liu Z. Zhang B. Cai J. Yao X. Zhang M.. (2023). Inter-bacterial mutualism promoted by public goods in a system characterized by deterministic temperature variation. Nat. Commun. 14 (1), 5394. doi: 10.1038/s41467-023-41224-7 37669961 PMC10480208

[B47] Zhao X.-y. Yang J.-j. Li S.-k. Lu X.-x. Li X. (2021). Research progress on lignin degradation mechanism and influencing factors during composting. Environ. Eng. 39, 128. doi: 10.13205/j.hjgc.202106019

[B52] Zhao G.-H. Yu Y.-L. Zhou X.-T. Lu B.-Y. Li Z.-M. Feng Y.-J. (2017). Effects of drying pretreatment and particle size adjustment on the composting process of discarded flue-cured tobacco leaves. Waste Manage. Res. 35, 534–540. doi: 10.1177/0734242x17690448 28190373

[B54] Zheng G. Chen T. Yu J. Gao D. Shen Y. Niu M.. (2015). Impact of composting strategies on the degradation of nonylphenol in sewage sludge. Ecotoxicol. (London England) 24, 2081–2087. doi: 10.1007/s10646-015-1558-x 26452367

[B55] Zhou M. Guo P. Wang T. Gao L. Yin H. Cai C.. (2017). Metagenomic mining pectinolytic microbes and enzymes from an apple pomace-adapted compost microbial community. Biotechnol. Biofuels 10, 198. doi: 10.1186/s13068-017-0885-y 28852421 PMC5568718

