# Supplementary material for: Microbial community succession of cow manure and tobacco straw composting
Source: Front Microbiomes. 2024 Feb 8;3:1301156. doi: 10.3389/frmbi.2024.1301156 (PMC12993546; doi:10.3389/frmbi.2024.1301156)
Supplement: Supplementary file 1 [file Table_1.doc]

SUPPLEMENTARY TABLE

Table S1. Physical-chemical parameters of the cow manure and tobacco straw compost along the composting process

|  | TN (g/kg) | TP (g/kg) | TK (g/kg) | Total organic carbon (g/kg) | NO_3_^-^-N | NH_4_^+^-N | C/N | Moisture content (%) | Full amount of nutrients (%) |
| --- | --- | --- | --- | --- | --- | --- | --- | --- | --- |
| NY425 | 13.4±0.84 | 4.53±0.43 | 16.5±2.06 | 320.4±17.4 | 1.55±0.755 | 40.1±5.17 | 23.9 | 59.7±4.28 | 34.43 |
| NY504 | 13.3±0.56 | 5.19±0.41 | 14.5±0.89 | 352.7±10.2 | 0.376±0.112 | 52.1±6.44 | 26.5 | 64.2±5.79 | 32.99 |
| NY510 | 14.3±2.69 | 5.24±0.52 | 16.9±0.92 | 344.1±9.36 | 1.37±0.547 | 47.2±4.59 | 24.1 | 43.6±3.66 | 36.44 |
| NY515 | 13.6±0.39 | 5.52±0.56 | 14.5±5.47 | 343.1±5.44 | 1.12±0.385 | 60.6±6.12 | 25.2 | 59.6±6.49 | 33.62 |
| NY521 | 13.2±3.45 | 5.28±0.78 | 14.6±2.48 | 327.9±5.47 | 1.07±0.351 | 46.3±4.22 | 23.1 | 52.4±4.57 | 33.08 |

TP, total phosphorus; TK, total potassium.
